# Neuroscience perspectives on security

**DOI:** 10.3389/fnhum.2014.00996

**Published:** 2014-12-09

**Authors:** Elena Rusconi, Kenneth C. Scott-Brown, Andrea Szymkowiak

**Affiliations:** ^1^Department of Security and Crime Science, University College LondonLondon, UK; ^2^Division of Psychology, Abertay UniversityDundee, UK; ^3^Department of Neurosciences, University of ParmaParma, Italy; ^4^School of Science, Engineering and Technology, Abertay UniversityDundee, UK

**Keywords:** security, deception detection, threat detection, crime science, neuroenhancement, applied neuroscience, applied psychology, military

Security issues have been under the spotlight on a daily basis since the 9/11 terrorist attacks to the Twin Towers, which—aired on live TV—were witnessed by millions of people around the globe. This has been accompanied by the increased availability (and leakage) of security information on the Internet, the increase in public awareness over related issues, and the surge of ethical debates on the possible ethical and legal consequences of “security states”; security has taken priority in political agendas, academic debates, and research funding—the security industry is thriving. Against this background, more and more academics are exploring ways to contribute to the debate, and to inform and influence security decision making. This is both a challenging and a rewarding enterprise and neuroscience promises game-changing innovations.

Security science however is a multidisciplinary field, where physics and engineering, computer science and biology, psychology and medicine, pharmacology and neuroscience, philosophy and jurisprudence, sociology and ethology can all bring valuable contributions to the table. Accordingly, in this Research Topic we have hosted relevant contributions from neuroscience and psychology experts but also dipped into other disciplines such as engineering, physics, computer science, crime science, jurisprudence, and sociology of science. We would like to thank all of the authors and the reviewers for their excellent contributions and their effort in spanning disciplinary boundaries. It is not easy to strike the right balance between expertise and accessibility, to explore a little further outside of our comfort niche and convey meaning to a multifaceted type of readership, such as the one that can be reached via open access and via Frontiers in Human Neuroscience in particular. We hope that our Research Topic will provide a useful contribution to the dialog among disciplines on security-related issues and also a successful example of how the—often artificial—disciplinary boundaries can be challenged.

Almost every aspect of security is inextricably connected with technology. One of the aspects where accelerated advancements have been witnessed in recent years is the incorporation of psychological and physiological measures via new technologies. Reviews of the area of biometrics, traditionally described as the identification of individuals (or their emotional states) using physiological and behavioral characteristics, such as finger prints, iris or retinal patterns, facial features, handwriting or typing on a keyboard (see Ahmad et al., 2013), to name but a few, and the uses of sophisticated imaging techniques, such as fMRI to detect indicators of deception (see Rusconi and Mitchener-Nissen, 2013; Vartanian et al., 2013), provide two representative examples of this. The ethical and legal aspects of the use of such technologies are widespread. One the one hand, the data gathered with such technology are challenging to process and interpret, so this bears the question as to how clearly experts can present their evidence to a jury in the context of criminal justice systems; on the other hand, findings based on the use of the technology are still far from being fully reliable, research based on laboratory experiments restricts the ecological validity of such measures, and the complexity and sensitivity of the technology makes it difficult to run trials outside the laboratory or even envisage real-world applications. Another question pertains to how transparent individuals and their internal (e.g., emotional, intentional, deceptive, etc.) states can ever be made, even with a fine-grained analysis of human behavior or characteristics, as individuals become aware of advancements in technologies to assess these. Drawing on the concept of measures and countermeasures—can human suspicious behavior and intent be camouflaged so well it is not traceable by the latest neuroscientific detection systems? It is not yet clear to which extent the sophistication of technology and human perception to assess human mental and behavioral activity is juxtaposed with the sophistication of individuals to evade these security measures. Further, fully successful detection systems would have human rights, policy making and social acceptance implications, a critical issue that has been clearly recognized (see Mitchener-Nissen, 2013; Rusconi and Mitchener-Nissen, 2013).

While the above methods investigate physiological or behavioral indices with technological means and algorithms, the use of human operators during incident or threat detection is still irreplaceable and critical to the security discourse (see Howard et al., 2013; Mendes et al., 2013; Stainer et al., 2013). This bears the question on how secure we actually are as both technology and humans are fallible in their decision making. It is, however, generally assumed that the output of visualization techniques such as CCTV and transmission x-rays can be appropriately assessed by trained individuals. CCTV operators are presented with large volumes of constantly updating visual information, and the navigation through this temporal and spatial data feed is very demanding. In transmission x-rays, the difficulty of complex image interpretation lies mostly in the superposition of several two-dimensional projections and the unusual views by which objects are seen in static images. To gather information about human performance in security image interpretation, diametrically opposite approaches can be adopted—from a classical hypothesis-driven experimental method to an *in situ* observational method reminiscent of a cognitive-ethological approach (Howard et al., 2013; Stainer et al., 2013). While technological improvements are being pursued to increase the efficiency of the screening process from an engineering and physics standpoint, these efforts may be hindered by the intrinsic limitations of the human visual perception system (see Mendes et al., 2013). Notably, to the extent that decisions are made by people, the assessment of potentially dangerous situations in a social environment is subjected to the limitations of the cognitive system that can be swayed or driven by appearances, biases, and previous experience (see Watkins, 2013; Woody and Szechtman, 2013). Of course, the same constraints will also apply to the decisions made by those individuals who actively engage in criminal activities (i.e., those who create breaches in security rather than help maintain it)—an awareness that seems yet to have been fully incorporated in evidence-based crime science (Bouhana, 2013).

Brain manipulation techniques such as Transcranial Magnetic Stimulation and transcranial Direct Current Stimulation may help overcome some of the intrinsic limitations of human security operators with their potential to augment human performance in a range of tasks (Levasseur-Moreau et al., 2013; Parasuraman and Galster, 2013). Although the state of the art may not be mature enough to allow for direct translations into the security field, it is of paramount importance that neuroscientists engage as early as possible with professionals from other disciplines to formulate critical appraisals of the larger-picture implications of any of the envisaged uses (Brunelin et al., 2013; Sehm and Ragert's, 2013). Arguably, rather than hinder or slow down scientific progress, these early multidisciplinary appraisals and interactions will help secure more public support and more resources for neuroscience research.

## Conflict of interest statement

The authors declare that the research was conducted in the absence of any commercial or financial relationships that could be construed as a potential conflict of interest.
